# Supplementing Ruminally Protected Lysine, Methionine, or Combination Improved Milk Production in Transition Dairy Cows

**DOI:** 10.3389/fvets.2022.780637

**Published:** 2022-03-25

**Authors:** Samy A. Elsaadawy, Zaohai Wu, Han Wang, Mark D. Hanigan, Dengpan Bu

**Affiliations:** ^1^State Key Laboratory of Animal Nutrition, Institute of Animal Sciences, Chinese Academy of Agricultural Sciences, Beijing, China; ^2^Department of Dairy Science, Virginia Tech, Blacksburg, VA, United States; ^3^Joint Laboratory on Integrated Crop-Tree-Livestock Systems of the Chinese Academy of Agricultural Sciences (CAAS), Ethiopian Institute of Agricultural Research (EIAR) and World Agroforestry Centre (ICRAF), Beijing, China; ^4^Hunan Co-Innovation Center of Safety Animal Production, Changsha, China

**Keywords:** periparturient cattle, amino acids, energy balance, methionine, β-hydroxybutyrate, milk, lysine, body condition score

## Abstract

The objectives of this study were to evaluate the effects of dietary supplementation of ruminally protected lysine (RPL), or methionine (RPM), and their combination (RPML) on the production efficiency of transition cows. A total of 120 pre-partum multiparous Holstein cows were assigned to four treatments based on previous lactation milk production, days (d) of pregnancy, lactation, and body condition score (BCS). Cows were fed a basal diet [pre-calving: 1.53 Mcal/kg dry matter (DM) and post-calving: 1.70 Mcal/kg DM] with or without supplemental ruminally protected amino acids (RPAA). Treatments were the basal diets without supplemental amino acids (CONTROL, *n* = 30), with supplemental methionine (RPM, pre-calving at 0.16% of DM and post-calving at 0.12% of DM, *n* = 30), with supplemental lysine (RPL, pre-calving at 0.33% of DM and post-calving at 0.24% DM, *n* = 30), and the combination (RPML, pre-calving at 0.16% RPM + 0.33% RPL of DM and post-calving at 0.12% RPM + 0.24 % RPL DM, *n* = 30). The dietary content of lysine was balanced to be within 6.157.2% metabolizable protein (MP)–lysine and that of methionine was balanced within 2.1–2.35% MP-methionine. Dry matter intake (DMI) was measured daily. Milk samples were taken on d 7, 14, and 21 days relative to calving (DRC), and milk yields were measured daily. Blood samples were taken on d −21, −14, −7 before expected calving and d 0, 7, 14, and 21 DRC. Data were analyzed using SAS software. There were significant Trt × time interactions (*P* < 0.01) for DMI pre- and post-calving period. The CON cows had lower DMI than RPM, RPL, and RPML, both pre-calving (*P* < 0.01) and post-calving periods (*P* < 0.01). Energy-corrected milk (*P* < 0.01), milk fat (*P* < 0.01), protein (*P* = 0.02), and lactose (*P* < 0.01) percentage levels were greater for RPM, RPL, and RPML cows compared to CON. Supplementing RPAA assisted in maintaining BCS post-calving than CON (*P* < 0.01). Blood concentrations of β-hydroxybutyrate decreased with RPM or RPL or the combination pre-calving (*P* < 0.01) and tended to decrease post-calving (*P* = 0.10). These results demonstrated that feeding RPL and RPM improved DMI and milk production efficiency, maintained BCS, and reduced β-hydroxybutyrate concentrations of transition cows.

## Introduction

Dairy cows undergo significant metabolic, physiological, and immunological changes during the transition period, which, if not managed correctly, will increase the occurrence of metabolic disorders during early lactation (1). Peri-parturient cows mobilize body fat to meet their energy requirements (2), and to meet protein or amino acid (AA) requirements as well, because dry matter intake (DMI) is insufficient to meet the cow's requirements. The negative balance of energy and AAs is the main reason for many metabolic diseases and problems, and its consequences cause reduced milk production, lower fertility, increased health disorders, and decreased animal efficiency in the long term.

Improving the AA profile for milk protein synthesis *via* dietary AA supplementation can alleviate intensive AA mobilization in dairy cows after parturition, thus sparing body protein reserves. Methionine (Met), lysine (Lys), and histidine (His) are generally considered limiting AAs in diets for high-producing cows (3–7). Metabolism of Met in the liver is very important; for instance, one-carbon metabolism has many roles in controlling the immunometabolic and growth during pregnancy, lactation, and the neonatal period in dairy cattle (8). Met is a source of the methyl donor S-adenosyl Met (9). Probably, the most important metabolic role of Met is that of a hepatic lipotropic agent promoting synthesis of very low–density lipoproteins (VLDL), which minimizes the accumulation of triacylglycerol (TAG) in the liver (10, 11). Carnitine is synthesized from Lys (carbon backbone) and Met (methyl group donor) (12), which is required for the oxidation of long-chain fatty acids (LCFAs) and the regulation of ketosis.

Transition dairy cows fed ruminally protected Lys (RPL) and ruminally protected Met (RPM) during both the pre- and post-calving periods had increased milk and milk protein production (13, 14), but this response is not universal (15). Pre- and post-calving supplementation of only RPM may be sufficient to elicit the response (16, 17). The inclusion rate of AAs in diets is varied. As an example, Lee et al. (15) supplied a combination of RPL and RPM to transition dairy cows either before calving (0.14% RPM + 0.24% RPL of DM) or post-calving (0.22% RPM + 0.36% RPL of DM) or throughout the peri-parturient period (15). Others fed 15 g/d of RPM to post-calving cows (14). Fehlberg et al. (18) supplied 0.54% and 0.40% of DM RPL during pre- and post-calving, respectively, to transition cows (19). Studies on beef cows supplied RPM (10 g/d to provide a 3.7 g/d of absorbed Met) (20) or at a 9 g/d (18). Therefore, in the current study, the dietary supplementation of RPL was balanced to have Lys within 6.15–7.2% metabolizable protein (MP)–Lys and Met to within 2.1–2.35% MP-Met.

Previous studies have been conducted to examine supplementation of one ruminally protected AA (RPAA) (in most cases, but not all) to low or medium energy transition diets, generally either in the pre-or post-partum periods, with only a few studies supplementing throughout the peri-parturient period (14, 15, 19, 21, 22). To our knowledge, no studies have been conducted to examine the effects of supplying a combination of RPAA to high-energy diets of transition cows. It was hypothesized that continual supply of either RPM or RPL to high energy diets fed to transition dairy cows would stimulate DMI, driven by the improvement of AA balance, leading to improve energy and MP balance and increased milk production. We also hypothesized that the improvement of lactation performance and health would be greatest when cows have consumed the combination of RPL and RPM. The objectives of this study were to assess the effects of the supplementation of RPL or RPM and the combination of high energy density diets on production efficiency and BHB concentrations of transition dairy cows.

The results have been presented in partial form during the 2021 Annual Meeting of American Dairy Science Association (ADSA), Abstract No. 245 “Supplementing ruminally protected methionine or lysine improved milk production in transition cows” (23, 24).

## Materials and Methods

### Experimental Design and Animals

The trial was conducted from December 2019 to March 2020 at a mega-dairy with a total capacity of 10,000 milking cows, AustAsia Dairy, Shandong, China. A total of 120 Holstein dairy cows were randomly allocated to eight groups [replicates (15 cows per each replicate)], and two replicates were assigned to each of the four experimental treatments (*n* = 30 cows per treatment): (1) CON cows received the basal diet without supplemental RPAA; (2) RPM received CON plus RPM; (3) RPL received CON plus RPL; and (4) RPML received CON plus RPM and RPL. RPM, RPL, and RPML all received the same amount of supplemental RPAAs.

A statistical power analysis was conducted before the trial using a two-tailed α of 0.05, a power of 0.95, and an expected effect size of 0.35 using G–Power 3.1 software (25). At that power, 25 cows per treatment were required in order to detect a minimum of about 6% difference in post-calving DMI between treatment groups. The experiment was conducted as a completely randomized design with treatments arranged in a 2 × 2 factorial. Cows were selected and assigned to treatment based on days (d) pregnant (250 ± 2 d, *P* = 0.84), previous lactation milk production (11,512 ± 1,837 kg; 305-d milk production, *P* = 0.90), parity (3.09 ± 1.56, *P* = 0.94), and body condition score (BCS; 3.58 ± 0.26, *P* = 0.86).

The cows were fed their respective diets starting 3 weeks (wk) (25.0 ± 3.31 d) before the expected calving to 3 wk post-calving (24.0 ± 3.31 d). Cows were fed total mixed rations (TMR) *ad libitum* four times per day at 0600, 1,200, 1,800, and 2,400 h and milked in the rotary milking parlor four times per day just before being fed, at ~6-h intervals. Feed offered was managed to achieve a target of 5% refusals. The composition, chemical analyses, and AA profiles of the diets are presented in (Tables 1–3).

**Table 1:** Ingredient composition of diets fed to Holstein cows during the transition period [close-up pre-calving diets (the last 3 wk before calving) to the early post-calving (the first 3 wk after calving)]^a^.

	**Close-up pre-calving diet**				**Early post-calving diet**			
**Ingredient (% DM)**	**CON**	**RPM**	**RPL**	**RPML**	**CON**	**RPM**	**RPL**	**RPML**
Corn silage^b^	32.4	32.4	32.4	32.4	27.4	27.4	27.4	27.4
Grass hay	8.7	8.7	8.7	8.7	—	—	—	—
Oats hay	25.3	25.3	25.3	25.3	5.6	5.6	5.6	5.6
Alfalfa hay^c^	—	—	—	—	17.9	17.9	17.9	17.9
Corn grain flaked^d^	—	—	—	—	5.3	5.3	5.3	5.3
Corn grain fine	10	10	10	10	17	17	17	17
Soybean meal^e^	4.4	4.4	4.4	4.4	6.9	6.9	6.9	6.9
Canola meal solvent^f^	7.4	7.4	7.4	7.4	2.7	2.7	2.7	2.7
Molasses cane^g^	—	—	—	—	1.9	1.9	1.9	1.9
Corn gluten meal^h^	—	—	—	—	1.7	1.7	1.7	1.7
Brewers grains^i^	7.4	7.4	7.4	7.4	5.6	5.6	5.6	5.6
Fresh cow premix^j^	—	—	—	—	5.8	5.8	5.8	5.8
Close-up premix^k^	4.17	4.17	4.17	4.17	—	—	—	—
ReaShure Choline^l^	0.12	0.12	0.12	0.12	0.10	0.10	0.10	0.10
Berga Fat 100^m^	—	—	—	—	0.7	0.7	0.7	0.7
Megalac^n^	—	—	—	—	0.8	0.8	0.8	0.8
Glycoline^o^	—	—	—	—	0.6	0.6	0.6	0.6
MetaSmart^p^	—	0.16	—	0.16	—	0.12	—	0.12
Lysipearl^q^	—	—	0.33	0.33	—	—	0.24	0.24

a *Close-up and early lactation periods (21 d to +21 relative to calving), Close-up cow rations were high energy (NE_L_ = 1.53 Mcal/kg of DM) with either rumen-protected Met or Lys or their combination RPML or without supplementation (RPM: 0.16% DM and RPL: 0.33% DM); early lactation rations: NE_L_ = 1.70 Mcal/kg DM, the dosage of ruminally protected amino acid was supplemented for the cows after calving (RPM: 0.12% DM and RPL: 0.24% DM). The cows were used for the statistical analysis are 27, 27, 29, and 25 for CON, RPM, RPL, and RPML, respectively*.

b *Corn silage contained 32% DM, 8.7% CP, and 38.52% aNDF*.

c *Alfalfa hay contained 91.3% DM, 21.4% CP, and 37.89% aNDF*.

d *Corn grain flaked contained 86.1% DM and 8.8% CP*.

e *Solvent soybean meal contained 86.7% DM and 47.5% CP*.

f *Canola meal Solvent contained 87.5% DM and 42.5% CP*.

g *Molasses sugarcane contained 60.5% DM and 4% CP*.

h *Corn gluten meal contained 91.8% DM and 64.6% CP*.

i *Brewers grains wet contained 22% DM and 31.67% CP*.

j *Fresh cow premix contained minerals (Na, Cl, Ca, P, Mg, K, and S), vitamins (A, D, and K), chelated minerals (Zn, Cu, Se, and Co), rumensin, and yeast*.

k *Close-up cow mineral premix: Na, Cl, Ca, P, Mg, K, and S*.

l *ReaShure Choline, encapsulated choline (Balchem Corporation, USA)*.

m *BergaFat100, rumen-protected fat (Berg and Schmidt Nutrition Sdn. Bhd., Malaysia)*.

n *Megalac (Volac Willmar feed ingredients Ltd., UK): bypass fats for ruminants providing extra energy without a carrier*.

o *Glucose precursors (VITALAC, France)*.

p *Rumen protected Met (MetaSmart, Adisseo, France)*.

q *Rumen protected Lys (Lysipearl, Kemin Industries, USA)*.

During the close-up and post-calving periods, cows were housed in a ventilated, four-row, free-stall barn (center feed alley with two rows of stalls on each side). After calving, cows were moved to the colostrum barn for 1 to 3 d, followed by relocation to the milking barn. Manure was removed by mechanical scraper four times per day at 0600, 1,200, 1,800, and 2,400 h. Sand bedding was groomed four times/d, and new sand was added two times/d at 0600 and 1,200 h. Cows had access to *ad libitum* water during the trial.

Temperature and humidity were measured during the pre-calving and post-calving periods (Humidity Detector PCE-HT 112, PCE Instruments Ltd., UK), and the temperature–humidity index (THI) was calculated according to the National Research Council (26).


$$\begin{array}{l} \text{THI}=\left(1.8\times \text{Tdb}+32\right)-\left(0.55-0.0055\times \text{RH}\right)\times \left(1.8\times \text{Tdb}-\;26\right), \end{array}$$

where Tdb-dry bulb temperature (°C) and RH - relative humidity(%).

### Ration Formulation

The isopropyl ester of 2-hydroxy-4-(methylthio)-butanoic acid (HMBi) was supplied in a dry powder form (MetaSmart, Adisseo, France). According to the manufacturer, the product contains 57% HMBi, with a Met equivalence of 78% and a ruminal absorption of 50%. Thus, each gram of the product provided 0.22 g of metabolizable Met. The RPL was provided as a dry powder containing 47.5% L-Lys monohydrochloride (3.2.3) with 70% bioavailability, according to the manufacturer. Thus, each gram of the product provided 0.33 g of metabolizable Lys-HCl (LysiPEARL, Kemin Industries, USA).

Rations were formulated using Cornell Net Carbohydrate and Protein System (CNCPS v. 6.5.5) as executed by AMTS.Cattle.Professional v. 4.7.2 (2016, AMTS LLC, USA) to meet or exceed requirements (Table 1). The dietary content of Met and Lys were balanced according to recommendations of CNCPS v6.5; for Lys to be within 6.15 to 7.2% of MP and Met within 2.1 to 2.35% of dietary MP—to achieve a Lys-to-Met ratio in the range of 2.5:1–3:1 for dairy cows. To achieve that, RPL was provided at rates of 0.33 and 0.24% of DM during the pre- and post-calving, respectively. Met was supplied during the close-up and post-calving periods at rates of 0.16 and 0.12% of DM, respectively (Tables 1–3). The close-up diet was formulated for close-up cows weighing 680 kg [body weight (BW)], with BCS of 3.5 and a predicted intake of 12.8 kg DM/d. The milking cow diet was formulated for fresh cows at 15 d in milk (DIM), weighing 608 kg, with BCS of 3.5, producing 33 kg milk/d with 3.4% crude protein (CP) and 4.2% milk fat, using a predicted DMI of 17.2 kg/d, and using the nutrition evaluation programs CNCPS.

RPAA was mixed with the feed additive premix using a mixer and then top-dressed on the TMR and mixed into the TMR four times/d during the trial using a Vertical Feed Mixer (Supreme International Limited, USA). A Premix is a blend of micronutrients where each nutrient component is prescaled and precision blended into a premix. Premixes include ingredients like vitamins, minerals, nucleotides, AAs, and other functional ingredients. Premix composition is detailed in Table 1.

### Sampling, Measurements, and Analysis

#### Feed and Physically Effective NDF

Feed offered and refused were measured daily for each treatment group. Diets and the major dietary ingredients (i.e., corn silage, alfalfa hay, corn grain, and soybean meal) were sampled and analyzed weekly and used to calculate nutrient concentrations. Feed samples were dried at 105°C for 4 h to determine the DM and stored at −20°C for further analysis. The dried feed samples were ground through a 1-mm screen before analysis using a Cyclotec 1093 Mill (Tecator 1093, Tecator AB, Höganäs, Sweden). Feed samples were further dried at 105°C for 2 h to determine the absolute DM. Chemical analyses of dry matter (DM), CP, ether extract (EE), neutral detergent fiber (aNDF), and acid detergent fiber (ADF) were performed using wet chemistry techniques at the State Key Laboratory of Animal Nutrition, Institute of Animal Sciences of Chinese Academy of Agricultural Sciences (CAAS), Beijing. China.

The content of CP (N × 6.25) in feed samples was analyzed using the macro-Kjeldahl nitrogen test (27) (method 984.13.4.09) with a Kjeltec digester 20 and a Kjeltec System 1026 distilling unit (Tecator AB). EE content was analyzed using a soxhlet HT6 apparatus (Tecator AB) according to method no. 920.39 (27). ADF and aNDF were analyzed according to (28) using alpha-amylase with the addition of sodium sulfite. Ash content was analyzed by incineration at 550°C, and the organic matter content was calculated by subtracting ash from 100. Non-fiber carbohydrates (NFCs) were calculated by difference according to the National Research Council (3).

Energy balance (EB), dietary energy density, and MP balance were calculated based on the actual consumed DMI per each treatment group of individual ingredients and their respective energy value and MP estimates from the CNCPS feed library.


$$\begin{array}{l} \text{Net energy intake }\left(\text{NEI}\right)=\text{daily DMI}\times \text{N}{\text{E}}_{\text{L}}\text{density of the diet} \end{array}$$

Requirements for NE were calculated as follows:


$$\begin{array}{l} \text{Net energy of maintenance }\left(\text{N}{\text{E}}_{\text{M}},\text{Mcal}/\text{d}\right)=\text{B}{\text{W}}^{0.75}\times \;0.080. \end{array}$$

The net energy of lactation (NE_L_, Mcal/d) was calculated according to the following:


$$\begin{array}{l} NE_{\text{L}}\;=\;(0.0929\times \text{fat\% }+\;0.0547\times \text{protein\% }+\;0.0395\\ \;\times \text{lactose\%})\times \text{milk production}. \end{array}$$

The net energy requirement for pregnancy (NE_Y_; Mcal/d) was calculated as

NE_Y_ = [(0.00318 × day of gestation – 0.0352) × (calf birth weight/45)]/0.218 for animals that were greater than 250 d pregnant, i.e., the pre-fresh group.

The sum of individual requirements was as follows:


$$\begin{array}{l} \text{N}{\text{E}}_{\text{REQ}}\left(\text{Mcal}/\text{d}\right)=\text{N}{\text{E}}_{\text{M}}+\text{N}{\text{E}}_{\text{L}}+\text{N}{\text{E}}_{\text{Y}} \end{array}$$

EB (Mcal/d) was calculated as follows:


$$\begin{array}{l} \text{EB}\left(\text{Mcal}/\text{d}\right)=\text{NEI}-\left(\text{N}{\text{E}}_{\text{L}}+\text{N}{\text{E}}_{\text{M}}+\text{N}{\text{E}}_{\text{Y}}\right).\\ \text{Supply}/\text{requirements}=\left[\text{NEI}/\left(\text{N}{\text{E}}_{\text{L}}+\text{N}{\text{E}}_{\text{M}}+\text{N}{\text{E}}_{\text{Y}}\right)\right]\times \;100. \end{array}$$

Energy-corrected milk (ECM; 3.5% fat) and fat corrected milk (FCM) were calculated according to (29):


$$\begin{array}{l} \text{ECM}\left(\text{kg}/\text{d}\right)=\left[0.323\times \text{ milk yield}\left(\text{kg}\right)\right]+\left[12.82\times \text{ fat yield}\left(\text{kg}\right)\right]\\ +\left[7.13\times \text{ protein yield }\left(\text{kg}\right)\right].\\ \text{FCM}\left(\text{kg}/\text{d}\right)=\left[0.4324\times \text{milk yield}\left(\text{kg}\right)\right]+\left[16.23\times \text{milk fat }\left(\text{kg}\right)\right]. \end{array}$$

Physically effective NDF (peNDF) of the TMR was determined weekly using a Penn State Particle Separator with three sieves: upper (19-mm pore size), middle (8-mm pore size), lower (4-mm pore size); it also has a pan. The peNDF content (DM bases) of the TMR was estimated by multiplying the NDF content of the feed by the percent of feed retained on each sieve (30).

#### Blood Samples and Analysis

Blood samples of ~5 ml were collected *via* coccygeal vessel from individual cows at 0700 h on d −21 (± 3.31 d), −14 (± 3.31 d), and −7 (± 3.31 d) before expected calving and d 0, 7 ± 3.31, 14 ± 3.31, and 21 ± 3.31 d post-calving. Blood β-hydroxybutyrate (BHBA)AQQ31 concentrations were measured immediately after collection of whole blood using the BHBCheck Plus (PortaCheck Inc., Moorestown, NJ, USA).

#### Milk Samples and Analysis

Milk samples were collected each milking session from individual cows on 7 ± 3.31, 14 ± 3.31, and 21 ± 3.31 DIM. Milk samples from each milking session from each cow were mixed and combined to make a representative milk sample (morning, afternoon, evening, and night; volume ratio of 3:3:2:2); each milk sample was preserved with bronopol-B2 preservative (D&F Control Systems Inc., Dublin, ON, Canada) and stored at 4°C with subsequent analysis for fat, protein, lactose, total solids (TSs), and milk urea nitrogen (MUN) by mid-infrared analysis (MilkoScan FT3; Foss-600, Foss Analytics, Hillerød, Denmark). Somatic cell count (SCC) was analyzed using a Somatic Cells Analyzer (Foss, Hillerød, Denmark). Daily milk production for individual cows was automatically recorded using parlor milk flow sensors, and data were transferred to the dairy herd management software [Delpro Software v5.4 (DeLaval Corporation, Sweden)]. Daily data were then summarized as average weekly data points for statistical analysis. Somatic cell score (SCS) was calculated by transforming SCC using the following equation (31):


$$\begin{array}{l} \text{SCS}=\text{log}2\left[\text{somatic cell counts}\left(\text{cells perml}\right)/100\right]+\;3. \end{array}$$

Milk N: The Kjeldahl method was used to measures the amount of nitrogen (N) in a milk sample. Milk protein contains ~15.65% N. Therefore, the milk protein concentration is calculated by multiplying the Kjeldahl N concentration by 6.38 (100 ÷ 15.65).

#### Body Condition Scoring

BCSs were assessed for individual cows once per week by the same two trained professionals from d 21 before expected calving until 3 wk post-calving. BCS was assessed using a five-point scale: 1 = extremely thin and 5 = extremely fat (3). Body condition scoring is a practical method of evaluating body energy stores in dairy cows. The BCS system included a combination of both visual appraisal and manual palpation to score individual cows. BCS evaluation is based on the visual appraisal of many-body points, commonly, between pins and hooks, transverse process, hooks, between the hooks, and tailhead to pins (3).

### Calculations and Statistical Analyses

One cow was excluded for calving too early at 265 d of pregnancy (RPML, *n* = 1). One cow was excluded from pre-calving due to vagal paralysis (RPML, *n* = 1). Three cows were excluded due to twins (CON, *n* = 1; PRM, *n* = 1; RPML, *n* = 1). The data were removed from 1 cow due to lameness problems (RPML, *n* = 1). Cows were excluded due to fracture (RPML, *n* = 1), milk fever (CON, *n* = 1), uterine prolapse (RPM, *n* = 1), or digestive problems (CON, *n* = 1; PRM, *n* = 1; RPL, *n* = 1). Therefore, the data for these cows were excluded from the statistical analysis. The cows were used for the statistical analysis are 27, 27, 29, and 25 for CON, RPM, RPL, and RPML, respectively.

Pre-calving and post-calving data were analyzed separately using a model containing the main effects of Met, Lys, and time and their interaction using PROC MIXED of SAS (v9.4, SAS Institute Inc., USA, 2013). The statistical model is as follows:


$$\begin{array}{l} {\text{Y}}_{\text{ijkl}}=\text{μ}+{\text{L}}_{\text{i}}+{\text{M}}_{\text{j}}+\text{L}{\text{M}}_{\text{ij}}+{\text{A}}_{\text{ijk}}+{\text{T}}_{\text{l}}+\text{L}{\text{T}}_{\text{il}}+\text{M}{\text{T}}_{\text{jl}}\\ +\text{LM}{\text{T}}_{\text{ijl}}+{\text{ε}}_{\text{ijkl}}, \end{array}$$

where Y_ijkl_ was the dependent, continuous variable; μ was the overall mean; L_i_ was the fixed effect of Lys; M_j_ was the fixed effect of Met; LM_ij_ was the interaction effect of Lys and Met; A_ijk_ was the random effect of the kth cow within the ijth treatment (combination of Lys and Met); T_l_ was the repeated, fixed effect of time (day); LT_il_ was the interaction effect of time and Lys; MT_jl_ was the interaction effect of time and Met; LMT_ijl_ was the interaction effect of Lys, Met, and time; and ε_ijkl_ was the residual error. Concentrations of β-hydroxybutyrate, milk yield, and composition were analyzed at different time points, and the covariance structure for the repeated measurements was modeled using the spatial power option. Computing the denominator degrees of freedom using the Kenward–Roger method (32) assessed the distribution of the residuals to determine normality and homoscedasticity. A log-transformation was used for the milk SCC variable to enhance the homogeneity of the distribution of residuals. The least-squares means were compared using the least significant difference, and statistical differences were declared significant at *P* ≤ 0.05. A tendency was considered at *P* > 0.05 to *P* ≤ 0.10.

## Results

### Barn Characteristics, Feed Particle Size

The average THI was 54.0 ± 4.0 for the close-up barn (min 41.8 and max 69.8) and 57.2 ± 3.9 for the fresh cow barn (min 43.3 and max 70.8). Particle size was as follows: 11.6 ± 0.73% of the pre-calving TMR DM was retained on the upper sieve; 38.4 ± 1.39% on the middle sieve, 17.9 ± 1.3% on the lower sieve, and 33.2 ± 1.5% on the bottom pan were collected. A 9.7 ± 0.70% of the post-calving TMR DM was retained on the upper; 39.9 ±1.40% on the middle sieve, 19.5± 1.28% on the lower sieve, and 31.1± 1.6% on the bottom pan were collected.

### Metabolizable Protein Balance

MP balance was positive in the pre-calving period for all treatments, but post-calving MP balances were negative across all dietary treatments. Diets supplied with RPM had greater MP supplies than other treatments during the close-up period supplying an average of 296, 79, and 47 g/d more MP than CON, RPL, and RPML diets, respectively (Table 2). Post-calving cows fed the CON diet had lower MP (656, 237, and 295 g/d) than RPML, RPM, and RPL cows, respectively (Table 2). The RPM and RPML diets provided ca. 0.20% units more Met to reach 2.59% of MP and provided 0.29% units more Lys to reach up to 7.0% of MP.

**Table 2:** Chemical composition and predicted metabolizable protein, Lys, and Met supplies of the diets fed to Holstein cows during the transition period [close-up pre-calving diets (the last 3 wk before calving) to the early post-calving (the first 3 wk after calving)]^a^.

	**Close-up pre-calving diet**				**Early post-calving diet**			
**Nutrients (% DM)**	**CON**	**RPM**	**RPL**	**RPML**	**CON**	**RPM**	**RPL**	**RPML**
NE_L_ (Mcal/kg DM)	1.53	1.53	1.53	1.53	1.70	1.70	1.70	1.70
NFC	35.9	35.9	35.9	35.9	40.3	40.3	40.3	40.3
Starch	18.9	18.9	18.9	18.9	25.8	25.8	25.8	25.8
Ether extract	3.8	3.8	3.8	3.9	4.3	4.3	4.3	4.3
NDF	36.8	36.8	36.8	36.8	28.6	28.6	28.6	28.6
CP	16.4	16.4	16.4	16.4	16.7	16.7	16.7	16.7
ADF	21.2	21.2	21.2	21.2	19.1	19.1	19.1	19.1
Ash	7.0	7.0	7.0	7.0	10.1	10.1	10.1	10.1
PeNDF	24.9	24.9	24.9	24.9	19.8	19.8	19.8	19.8
Forage%	63.3	63.0	63.3	63.0	52.5	52.5	52.5	52.3
**Metabolizable protein, Lys, and Met supply**								
DM%	49.4	49.4	49.4	49.5	59.5	59.5	59.5	59.6
RDP (% DM)	11.06	11.06	11.06	11.08	11.28	11.28	11.28	11.29
RUP (% DM)	5.29	5.29	5.29	5.29	5.42	5.42	5.42	5.42
RDP supplied (g/d)^*^	1,244	1,466	1,414	1,436	1,929	2,100	2,150	2,392
RUP supplied (g/d)^*^	570	763	707	736	948	1,111	1,149	1,404
MP supplied (g/d)^*^	1,177	1,473	1,394	1,426	1,862	2,099	2,157	2,518
MP balance (g/d)^*^	328	360	304	328	−524	−344	−299	−24
MP from bacteria (g/d)^*^	741	883	846	857	1,065	1,166	1,186	1,330
MP from RUP (g/d)^*^	436	590	548	569	775	913	949	1,167
LYS (g)^*^	83	102	103	105	125	139	151	177
Lys (% MP)^*^	7.03	6.91	7.4	7.37	6.7	6.62	6.99	7.01
Met (g/d)^*^	28.5	40.4	33.4	38.9	43.8	54.5	50.2	64.4
Met (% MP)^*^	2.43	2.75	2.4	2.73	2.36	2.59	2.33	2.56
Lys: Met^*^	2.9	2.52	2.8	2.7	2.84	2.55	3.0	2.74

a *Close-up and early lactation periods (21 d to +21 relative to calving), Close-up cow rations were high energy (NE_L_ = 1.53 Mcal/kg of DM) with either rumen-protected Met or Lys or their combination RPML or without supplementation (RPM: 0.16% DM and RPL: 0.33% DM); early lactation rations: NE_L_ = 1.70 Mcal/kg DM, the dosage of ruminally protected amino acid was supplemented for the cows after calving (RPM: 0.12% DM and RPL: 0.24% DM). The cows were used for the statistical analysis are 27, 27, 29, and 25 for CON, RPM, RPL, and RPML, respectively*.

* *Calculated based on the actual consumed DMI using CNCPS v. 6.5.5 as executed by AMTS.Cattle.Professional v. 4.7.2 (2016, AMTS LLC)*.

**Table 3:** Duodenal flows of the digestible indispensable amino acids (IAA) predicted for diets fed during the transition period by the Cornell Net Carbohydrate and Protein System [(CNCPS) v. 6.5.5]^a^.

	**Close-up pre-calving diets**							
	**CON**		**RPM**		**RPL**		**RPML**	
**(IAA)**	**AA flow**,	**MP**,	**AA flow**,	**MP**,	**AA**	**MP**,	**AA flow**,	**MP**,
	**g/d**	**%**	**g/d**	**%**	**g/d**	**%**	**g/d**	**%**
ARG	95.9	6.61	118.4	6.55	112.1	6.55	114.2	6.52
HIS	39.0	2.60	48.2	2.59	45.6	2.59	46.5	2.58
ILE	79.2	5.23	97.4	5.16	92.4	5.17	94.0	5.15
LEU	118.5	7.66	147.0	7.65	139.1	7.63	11.7	7.61
LYS	105.4	7.03	129.1	6.91	130.0	7.40	132.3	7.37
MET	39.8	2.43	59.2	2.75	46.4	2.40	57.1	2.73
PHE	77.7	4.99	96.0	4.96	90.9	4.95	92.6	4.94
THR	74.3	5.01	91.4	4.95	86.7	4.96	88.2	4.93
TRP	23.4	1.43	28.8	1.41	27.3	1.41	27.8	1.41
VAL	87.2	5.64	107.3	5.58	101.8	5.58	103.6	5.56
	**Early post-calving diets**							
	**CON**		**RPM**		**RPL**		**RPML**	
**(IAA)**	**AA flow**,	**MP**,	**AA flow**,	**MP**,	**AA**	**MP**,	**AA flow**,	**MP**,
	**g/d**	**%**	**g/d**	**%**	**g/d**	**%**	**g/d**	**%**
ARG	140.6	6.33	157.2	6.29	161.0	6.27	185.8	6.21
HIS	58.6	2.57	65.7	2.56	67.3	2.55	77.8	2.54
ILE	117.8	5.09	131.4	5.05	134.5	5.03	154.7	4.98
LEU	192.7	8.21	216.7	8.21	222.2	8.20	258.0	8.20
LYS	154.5	6.70	171.9	6.62	185.2	6.99	216.0	7.01
MET	58.7	2.36	76.1	2.59	67.0	2.33	89.5	2.56
PHE	119.1	5.04	133.4	5.02	136.3	5.01	158.0	4.98
THR	110.1	4.86	122.9	4.81	125.9	4.80	144.9	4.75
TRP	34.0	1.37	37.8	1.35	38.7	1.35	44.4	1.33
VAL	130.0	5.52	145.2	5.48	148.7	5.47	171.3	5.42

a *Close-up and early lactation periods (21 d to +21 relative to calving), Close-up cow rations were high energy (NE_L_ = 1.53 Mcal/kg of DM) with either rumen-protected Met or Lys or their combination RPML or without supplementation (RPM: 0.16% DM and RPL: 0.33% DM); early lactation rations: NE_L_ = 1.70 Mcal/kg DM, the dosage of ruminally protected amino acid was supplemented for the cows after calving (RPM: 0.12% DM and RPL: 0.24% DM). The cows were used for the statistical analysis are 27, 27, 29, and 25 for CON, RPM, RPL, and RPML, respectively*.

### Dry Matter Intake

**Figure 1:**
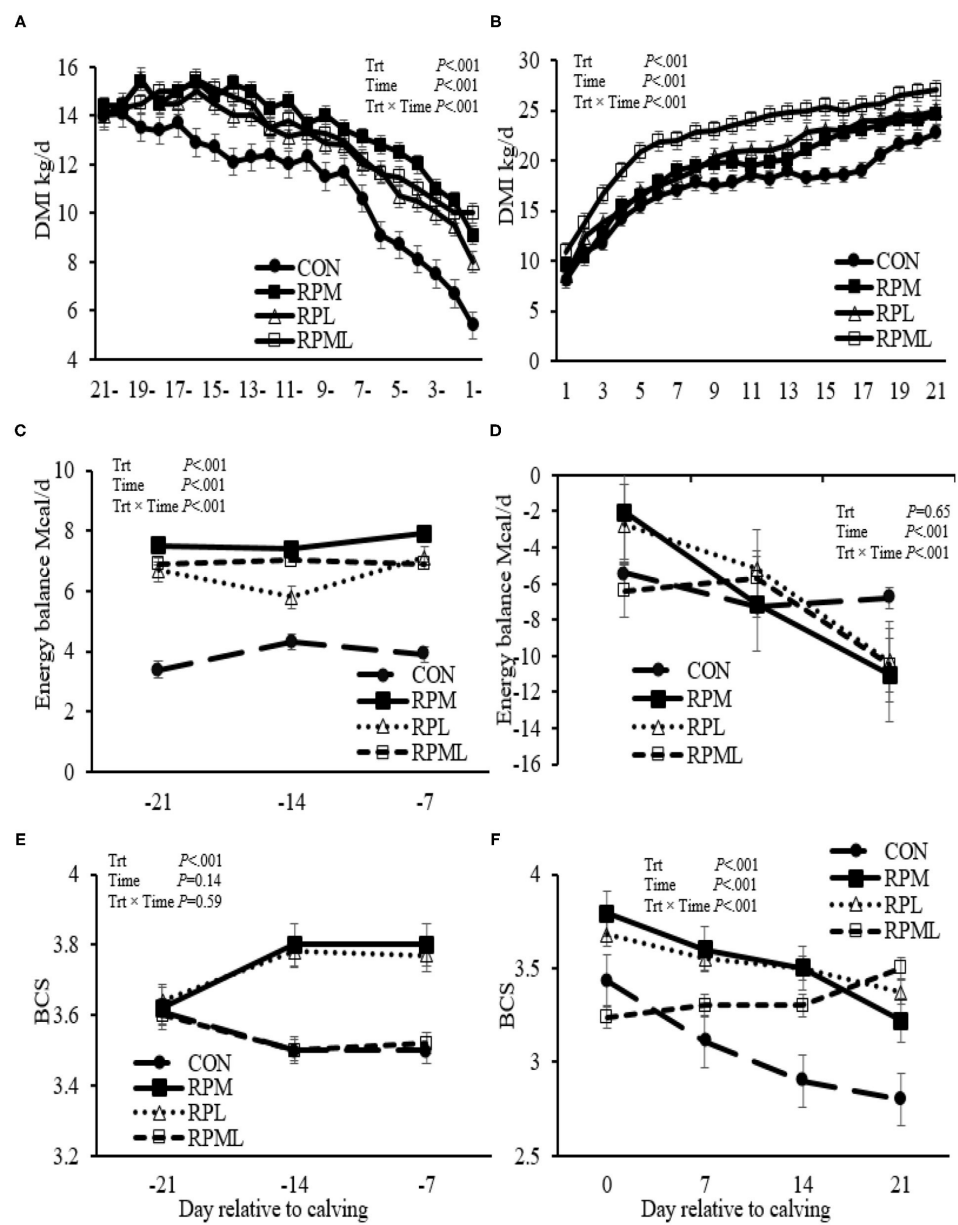
Effect of ruminally protected Lys and Met supplementation on dry matter intake, energy balance (EB; Mcal/d), and body condition score of Holstein dairy cows during the transition period. Values are means; standard errors are represented by vertical bars.

There were significant Trt × Time interactions for DMI (*P* < 0.001), but DMI of the RPAA treatments was always greater (*P* < 0.001) than the CON group pre-calving (13.3 vs. 11.2 kg/d) and post-calving (20.6 vs. 17.4 kg/d) (Table 4, Figures 1A,B). DMI was higher for cows that consumed RPM than those cows receiving either RPL or RPML pre-calving (*P* < 0.001; 13.6 vs. 12.9, 13.2 kg/d, respectively). RPML cows had greater DMI compared with RPM or RPL post-calving (*P* < 0.001, 22.6 vs. 19.4, 19.8 kg/d, respectively). Cows fed the RPL consumed more DMI than cows fed RPM post-calving (*P* < 0.001, 19.8 vs. 19.4 kg/d, Table 4).

**Table 4:** Effect of supplementation of ruminally protected Met and Lys on DMI, BCS, Energy Balance, and BHB of Holstein cows during the transition period.

	**Treatment** ^**1**^					***P*** **-value**						
**Variable**	**CON**	**RPM**	**RPL**	**RPML**	**SEM^**2**^**	**Met^**3**^**	**Lys^**4**^**	**Met**	**Time^**6**^**	**Time**	**Time**	**Time**
								**×**		**×**	**×**	**×**
								**Lys^**5**^**		**Met**	**Lys**	**Met**
												**×**
												**Lys^**7**^**
												
**Pre-calving period** (Average 3 wk pre-calving period)												
DMI (kg/d)	11.2^d^	13.6^a^	12.9^c^	13.2^b^	0.24	<0.001	<0.001	<0.001	<0.001	0.012	<0.001	<0.001
Energy balance (Mcal/d)	3.87^d^	7.58^a^	6.54^c^	6.97^b^	0.03	<0.001	<0.001	<0.001	<0.001	0.010	<0.001	<0.001
BCS	3.52^b^	3.83^a^	3.77^a^	3.54^b^	0.04	0.362	0.665	<0.001	0.144	0.671	0.563	0.273
BCS Change	0.14^b^	0.06^b^	0.08^b^	0.33^a^	0.09	0.081	0.011	0.031	0.365	0.513	0.141	0.201
BHB (mmol/L)	0.91^a^	0.66^c^	0.74^b^	0.66^c^	0.029	<0.001	0.004	0.007	<0.001	<0.001	0.313	<0.001
**Post-calving period** (Average 3 wk post-calving period)												
DMI (kg/d)	17.4^d^	19.4^c^	19.8^b^	22.6^a^	0.22	<0.001	<0.001	<0.001	<0.001	0.005	0.002	<0.001
Energy balance (Mcal/d)	6.50	6.75	6.10	7.52	0.81	0.353	0.833	0.511	<0.001	0.161	0.033	<0.001
BCS	2.94^c^	3.43^ab^	3.47^a^	3.35^b^	0.05	<0.001	<0.001	<0.001	<0.001	0.003	<0.001	<0.001
BCS Change	0.63^a^	0.58^b^	0.31^c^	0.25^d^	0.08	0.080	<0.001	0.092	<0.001	<0.001	<0.001	<0.001
BHB (mmol/L)	1.50	1.28	1.27	1.08	0.12	0.109	0.082	0.863	<0.001	0.092	0.991	0.541

a,b,c,d *Mean values with different superscripts in the same row were significantly different (P <0.05)*.

1 *Close-up and early lactation periods (21 d to +21 relative to calving), Close-up cow rations were high energy (NE_L_ = 1.53 Mcal/kg of DM) with either rumen-protected Met or Lys or their combination RPML or without supplementation (RPM: 0.16% DM and RPL: 0.33% DM); early lactation rations: NE_L_ = 1.70 Mcal/kg DM, the dosage of ruminally protected amino acid was supplemented for the cows after calving (RPM: 0.12% DM and RPL: 0.24% DM). The cows were used for the statistical analysis are 27, 27, 29, and 25 for CON, RPM, RPL, and RPML, respectively. Value is the average 3 wk of closeup for the pre-calving period and 3 wk of early lactation for the post-calving period*.

2 *Standard error means of all treatments*.

3 *Met = effect of methionine*.

4 *Ly s = effect of lysine*.

5 *Interaction of Met × Lys*.

6 *Time = effect of time: d −21, −14, −7, 0, 3, 7, 14, and 21 relatives to calving*.

7 *Interaction of Time × Met × Lys*.

### Effects on Energy Balance and Body Condition Score

There was an interaction of Trt × Time for EB, but the supplemental cows were always had greater EB than those cows fed an unsupplemented diet. EB was higher for RPAA cows than cows receiving the CON diet pre-calving (*P* <0.001; 7.03 vs. 3.87 Mcal). There was no difference between treatments in EB post-calving (*P* = 0.65). There was an interaction of Trt × Time for EB post-calving (*P* <0.01), with cows fed CON diet had higher EB at wk 3 (6.85 Mcal) compared with that fed RPM, RPL, and RPML of 11.07, 10.32, and 10.49 Mcal, respectively (*P* < 0.05) (Table 4, Figures 1C,D).

There was a Trt × Time interaction for BCS post-calving (*P* < 0.01); cows that received the RPML diet had greater BCS at wk 3 (3.49 units) compared with those in RPM and CON (3.22 and 2.79 units, respectively; *P* < 0.05) and a tendency for increase BCS compared with those cows in RPL (3.37 units; *P* = 0.12). BCS was improved by RPAA supply post-calving (*P* < 0.01, 3.42 vs. 2.94 units). Time had a strong tendency for increase BCS pre-calving (*P* = 0.14); time affected BCS post-calving (*P* < 0.001; Table 4, Figures 1E,F). In the contrast analysis conducted for comparing the supplemented AA to the CON, the results indicated that supplemental-AA cows had a smaller BCS change than cows fed CON diet post-calving (*P* < 0.01; −0.21 vs. −0.63 units). There was no difference between treatments in BCS change pre-calving (*P* > 0.10).

### Effect on Blood β-Hydroxybutyrate Concentrations

**Figure 2:**
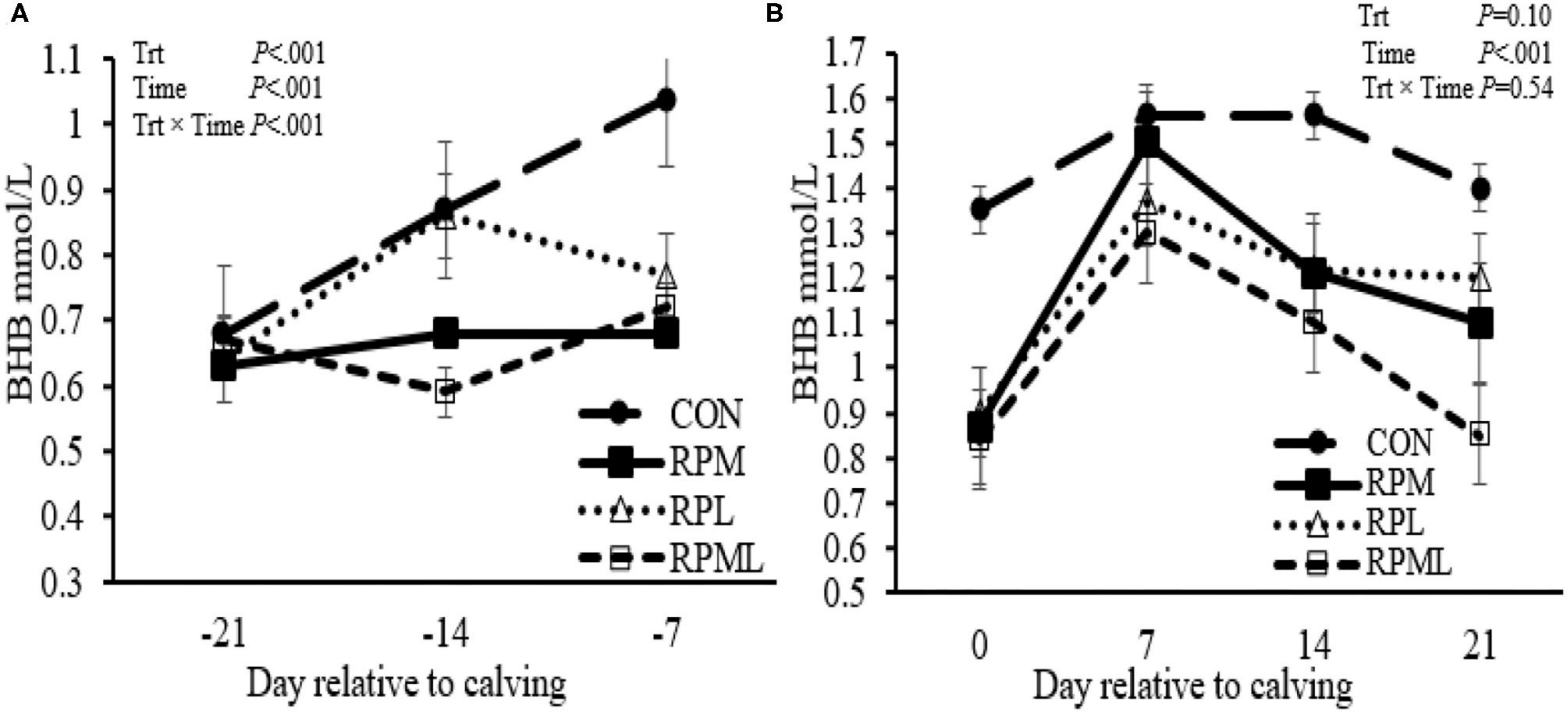
Effect of ruminally protected Lys and Met supplementation on blood β-hydroxybutyrate concentration of Holstein dairy cows during the transition period. Values are means; standard errors are represented by vertical bars.

Concentrations of blood BHBA decreased with RPM, RPL, or the combination pre-calving (0.69 vs. 0.91 mmol/L; *P* < 0.01), and there was a strong tendency for a decrease post-calving (1.21 vs. 1.50 mmol/L; *P* = 0.10) compared to unsupplemented cows (Table 4, Figures 2A,B). There was an interaction of Trt × Time for BHBA pre-calving (*P* < 0.01), in which CON cows had higher BHBA concentrations at wk −1 (1.04 mmol/L) compared with that fed RPM, PRL, and RPML (0.68, 0.77, and 0.72 mmol/L, respectively; *P* < 0.001). There was no Trt × Time interaction post-calving (*P* = 0.54) (Table 4, Figure 2A).

### Effects on Milk Production and Composition

**Figure 3:**
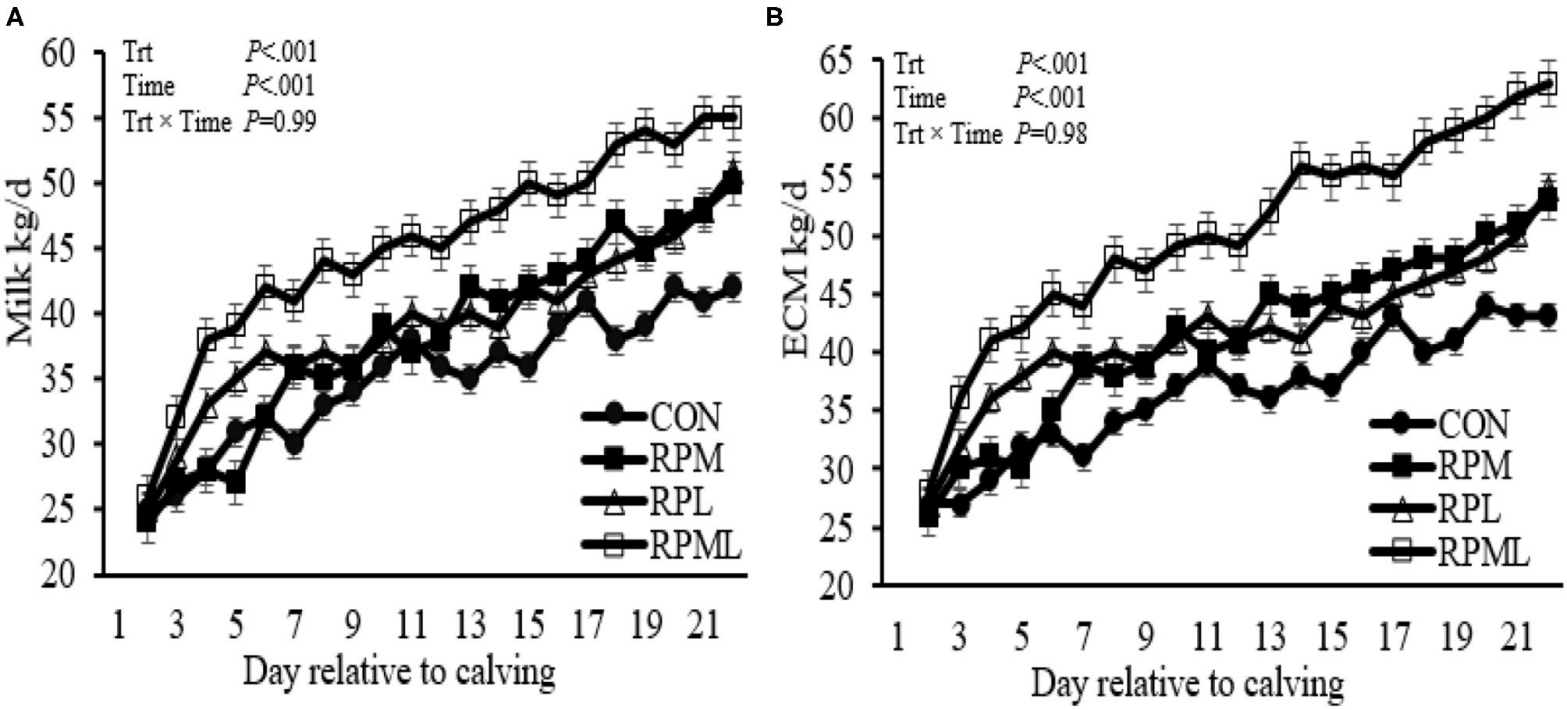
Effect of ruminally protected Lys and Met supplementation on milk production, energy-corrected milk yield of Holstein dairy cows during the transition period. Values are means; standard errors are represented by vertical bars.

**Figure 4:**
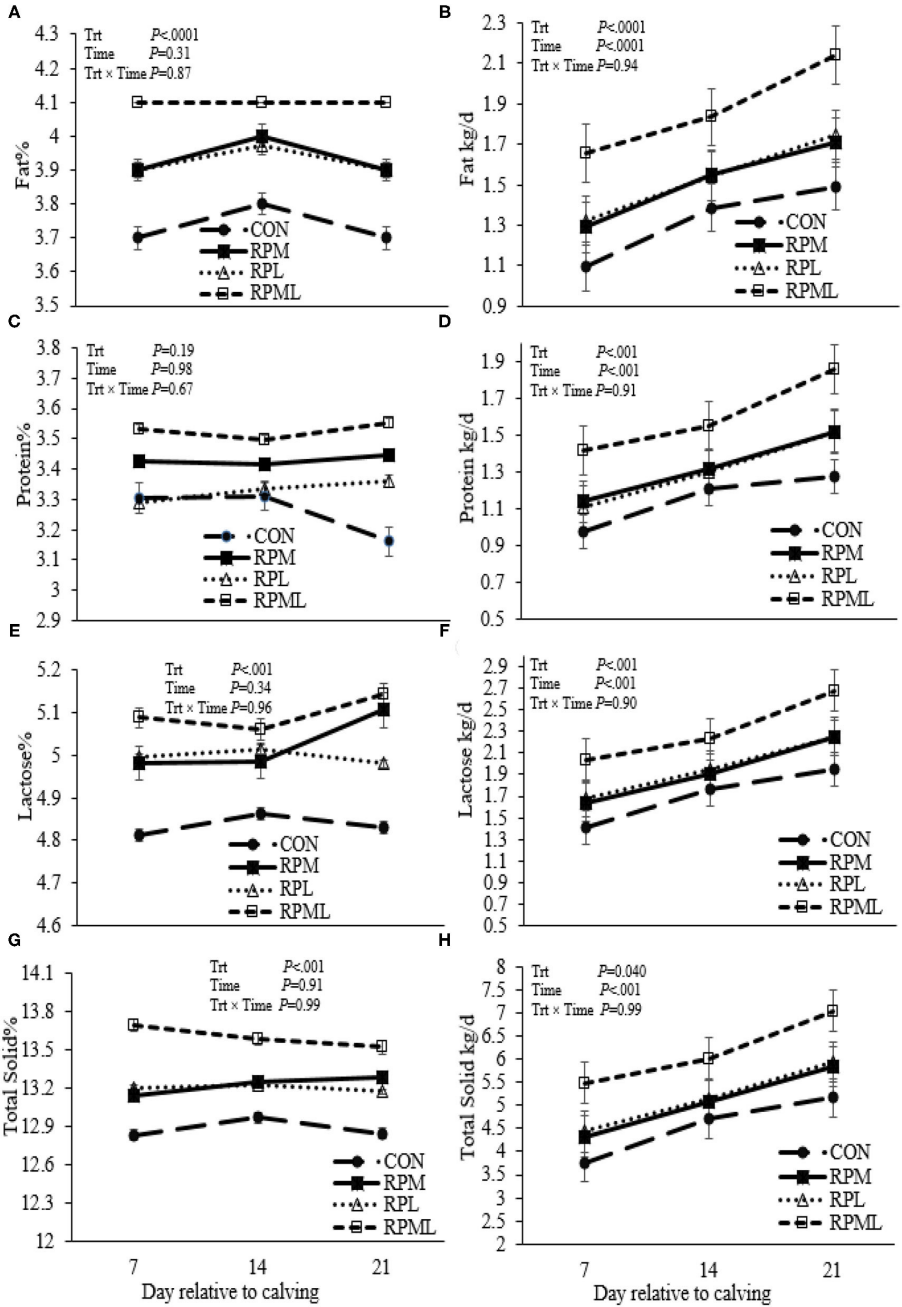
Effect of ruminally protected Lys and Met supplementation on some milk components and yields of Holstein dairy cows during the transition period. Values are means; standard errors are represented by vertical bars.

**Figure 5:**
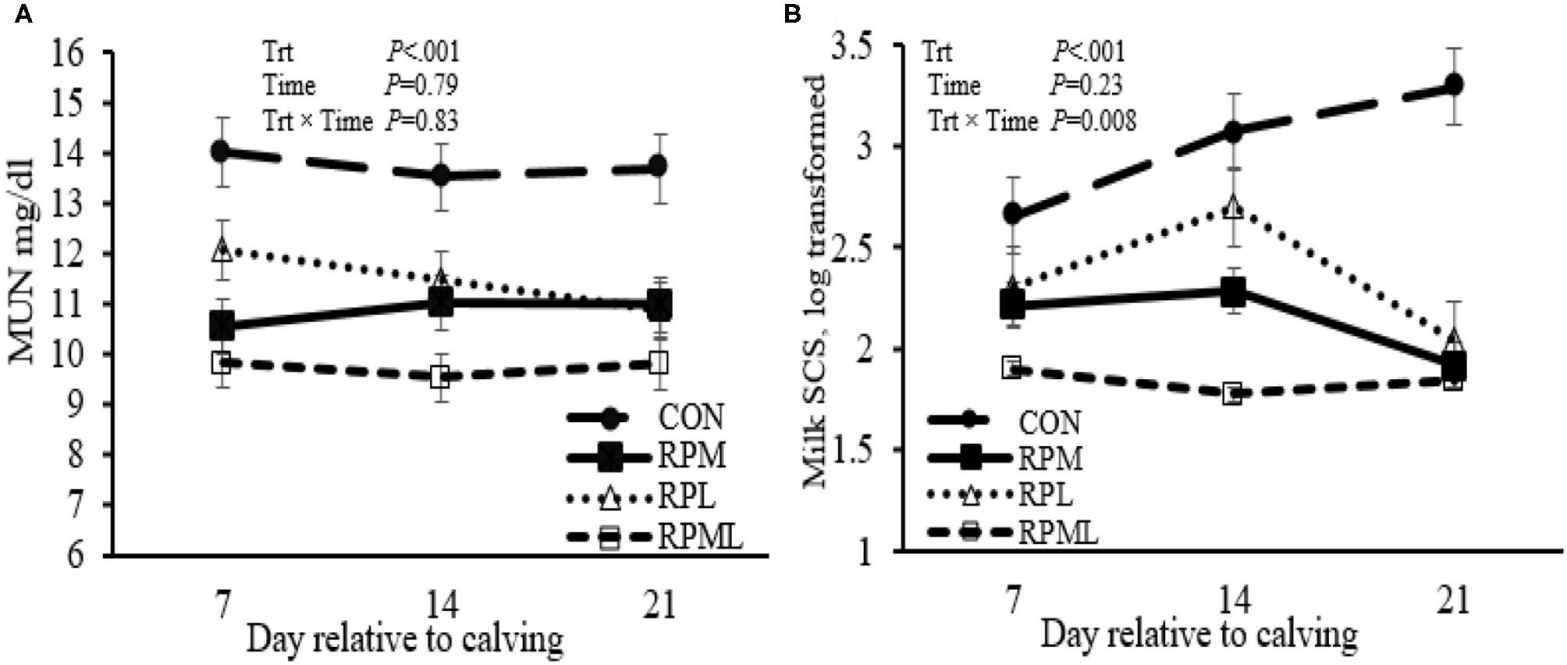
Effect of ruminally protected Lys and Met supplementation on MUN and somatic cell count of Holstein dairy cows during the transition period. Values are means; standard errors are represented by vertical bars.

Milk yield was greater (*P* < 0.05; 41.1 vs. 35.2 kg/d), ECM yield (*P* < 0.05; 44.4 vs. 36.5 kg/d) for cows fed RPAA was compared with the CON (Table 5, Figures 3A,B). Milk yield and ECM were not different between RPM and RPL treatment (*P* > 0.05). ECM yield was higher for cows that were fed RPML compared with those cows in RPM and RPL (*P* < 0.05; 50.2 vs. 41.5 kg/d). Milk fat (4.0 vs. 3.7%), crude protein (3.43 vs. 3.26%), lactose (5.4 vs. 4.8%), and TS (13.3 vs. 12.88%) contents and yield were greater for supplemented cows compared with CON (*P* < 0.05) (Table 5, Figures 4A–H). MUN of the CON group was always greater (*P* < 0.01) than RPAA treatments (9.7 vs. 13.74, 10.85, and 11.47 mg/dl) for CON, RPM, RPL, and RPML, respectively (Table 5, Figure 5A). There was a Time × Trt interaction for SCC (*P* = 0.008), with the CON group having greater SCC at wk 3 (3.47 log-transformed) compared with RPM, PRL, and RPML (3.25, 3.21, and 3.22 log-transformed, respectively; *P* < 0.05; Table 5, Figure 5B).

**Table 5:** Effect of supplementation of ruminally protected Met and Lys on milk yield and composition during 3 wk post-calving in Holstein dairy cows.

	**Treatment** ^**1**^					***P*** **-value**						
**Variable**	**CON**	**RPM**	**RPL**	**RPML**	**SEM^**2**^**	**Met^**3**^**	**Lys^**4**^**	**Met**	**Time^**6**^**	**Time**	**Time**	**Time**
								**×**		**×**	**×**	**×**
								**Lys^**5**^**		**Met**	**Lys**	**Met × Lys^**7**^**
**Milk production (kg/d)**												
Milk yield	35.2^c^	38.5^b^	39.2^b^	45.5^a^	1.09	<0.001	<0.001	0.160	<0.001	0.412	0.220	0.990
ECM	36.5^c^	41.3^b^	41.7^b^	50.2^a^	1.26	<0.001	<0.001	0.124	<0.001	0.373	0.151	0.985
FCM	36.7^c^	41.2^b^	41.9^b^	50.1^a^	1.25	<0.001	<0.001	0.136	<0.001	0.491	0.203	0.961
**Milk composition (%)**												
Fat	3.74^c^	3.94^b^	3.93^b^	4.11^a^	0.05	0.021	0.090	0.791	0.311	0.960	0.740	0.875
Protein	3.26^c^	3.43^b^	3.33^c^	3.53^a^	0.05	0.004	0.092	0.752	0.982	0.683	0.631	0.672
Lactose	4.83^c^	5.02^b^	5.00^b^	5.10^a^	0.03	<0.001	<0.001	0.115	0.341	0.122	0.731	0.961
Total solid	12.88^c^	13.23^b^	13.20^b^	13.60^a^	0.09	0.022	0.052	0.790	0.883	0.931	0.685	0.822
**Milk composition yield (kg/d)**												
Fat	1.32^c^	1.51^b^	1.54^b^	1.88^a^	0.05	<0.001	<0.001	0.131	<0.001	0.570	0.265	0.943
Protein	1.15^c^	1.32^b^	1.31^b^	1.61^a^	0.04	<0.001	<0.001	0.146	<0.001	0.225	0.130	0.915
Lactose	1.71^c^	1.94^b^	1.96^b^	2.32^a^	0.05	<0.001	<0.001	0.154	<0.001	0.201	0.126	0.902
Total solid	4.55^c^	5.09^b^	5.18^b^	6.19^a^	0.15	<0.001	<0.001	0.213	<0.001	0.080	0.251	0.991
MUN (mg/dl)	13.74^a^	10.85^b^	11.47^b^	9.71^c^	0.32	<0.001	<0.001	0.080	0.790	0.522	0.740	0.833
Milk SCS (log-transformation)^8^	3.01^a^	2.14^bc^	2.35^b^	1.84^c^	0.13	<0.001	<0.001	0.291	0.233	0.495	0.781	0.008
Feed efficiency ECM:DMI	2.11	2.14	2.10	2.21	0.06	0.263	0.644	0.554	<0.001	0.514	0.062	0.004
Nitrogen Efficiency (N milk/N feed)	39.7	41.1	39.4	42.4	1.43	0.114	0.765	0.541	<0.001	0.305	0.122	0.001

a,b,c *Mean values with different superscripts in the same row were significantly different (P < 0.05)*.

1 *Close-up and early lactation periods (21 d to +21 relative to calving), Close-up cow rations were high energy (NE_L_ = 1.53 Mcal/kg of DM) with either rumen-protected Met or Lys or their combination RPML or without supplementation (RPM: 0.16% DM and RPL: 0.33% DM); early lactation rations: NE_L_ = 1.70 Mcal/kg DM, the dosage of ruminally protected amino acid was supplemented for the cows after calving (RPM: 0.12% DM and RPL: 0.24% DM). The cows were used for the statistical analysis are 27, 27, 29, and 25 for CON, RPM, RPL, and RPML, respectively. Value is the average 3 wk of early lactation for the post-calving period*.

2 *Standard error means of all treatments*.

3 *Met = effect of methionine*.

4 *Lys = effect of lysine*.

5 *Interaction of Met × Lys*.

6 *Time = effect of time: d −21, −14, −7, 0, 3, 7, 14, and 21 relatives to calving day*.

7 *Interaction of Time × Met × Lys*.

8 *Milk somatic cell score (SCS)*.

### Feed Efficiency, Nitrogen Efficiency

There were no effects of AA supply on feed efficiency (ECM/DMI; *P* = 0.63) or nitrogen efficiency (feed N/milk N; *P* = 0.42). There was a time effect for feed and nitrogen efficiency (*P* < 0.05; Table 5). There was a Time × Trt interaction for feed efficiency at wk3 (*P* = 0.04), in which cows fed RPM and RPL had higher feed efficiency (*P* = 0.05; 2.45 and 2.41 ECM/DMI, respectively).

## Discussion

### Metabolizable Protein Balance

In the current study, the greater MP was supplied from RPAA diets, which was driven by increased DMI to sustain greater milk yield, improved EB, and carried over to subsequent post-calving performance. This assisted in the replenishment of body condition for cows fed RPML and RPL but not cows fed RPM. Overall, our study's post-calving MP balance in RPM cows was (−344 g/d) compared with a −617 g/d (33). The differences between our study and the prior study could have been partially associated with the stimulation of DMI (4.3 kg/d) greater in the current study than others (33). In addition, in our study, pre-calving diets provided with HMBi, RPL, and RPML delivered an equal amount of MP-Lys and 11 g more of MP-Met than the CON diet; cows that received an AA supply had an extra ca. 30 g of MP-Lys and ca. 12 g of MP-Met post-calving. In the work of Osorio et al. (33), cows that received HMBi had similar amounts of MP-Lys and an additional 7 g of MP-Met than the CON pre-calving, HMBi cows received an extra ca. 15 g of MP-Lys and ca. 11 g of MP-Met during the post-calving period. Similar to our results, they achieved an estimated increase of 9 g/d absorbed Met over the CON diet by supplying a 40.8 g/d HMBi (34).

### Dry Matter Intake

In the current study, stimulating DMI resulted in greater energy and total MP intake, which supported greater production and spared BW loss, attributed mainly to increased indispensable AAs (IAA) efficiency. In addition, stimulation of carnitine synthesis has been shown to increase DMI 1 to 3 g/d carnitine in lactating Holstein diets (35). Meyer et al. (36) showed that an L-carnitine supplementation of 25 g of rumen-protected L-carnitine per cow/d did not affect the DMI of transition dairy cows. However, the increased DMI in this study in response to Lys and Met was greater than reported by (35); thus, it is unlikely that carnitine is the sole contributor. Furthermore, other factors such as changes in metabolism, hormone concentrations, AA balance, and inflammatory and neural control may have affected DMI in the transition dairy cows.

The mechanism relating to AA and DMI has been shown in non-ruminants to impact food intake. There is considerable evidence for neural control of food intake in response to imbalanced-AA diets (37, 38). Our study found a 23% decrease in DMI for CON cows fed diets deficient in multiple IAA (−21.8 g MP-AA, equal to a 14.6% AA-deficient rate) as compared to cows fed balanced-IAA diets. Gietzen et al. (37) reported that food intake was decreased 55% for rats fed threonine (THR) deficient diets (12.5% THR deficient rate), similar to the current study. So, one might conclude that the mechanism may be contributing to decreasing DMI in cows fed imbalanced-IAA diets. In our study, a higher 23% DMI and 27.3% milk yield was observed in cows receiving a balanced-IAA diet than those cows that were fed multiple IAA-deficient diets (a 15.4% THR-deficient rate). In comparison, a 12.5% THR deficiency was required to cause a 55% decrease in food intake in rodents (37), which was similar to the AA deficiency in the prior study. It appears that IAA-deficiency in ruminants may have significant effects on DMI, and the reduction in DMI only appears when the IAA-deficiency is relatively large as compared to rodents. The mechanisms underlying the control of the feeding response to the dietary model remain not fully understood, and more research is needed.

The rate of free fatty acid oxidation in the liver also appears to regulate feed intake in ruminants. Allen and Piantoni (39) reported that hepatic oxidation of non-esterified fatty acid (NEFA) stimulated brain satiety centers *via* the hepatic vagal nerve to suppress intake. It is possible that liver concentrations of fatty acid or fatty acid–derived metabolites such as BHBA are being sensed rather than the oxidation rate (39); in either case, they were exporting more fatty acid as VLDL would lead to less oxidative stress in the liver and avoid intake suppression, which partially may explain the higher DMI for cows fed RPAA diets in the present study.

Increased DMI was generally (13, 40) but not universally (19) observed in response to the provision of RPL before calving. Supply RPL after calving also resulted in greater DMI when consumed pre-calving (13, 19, 40), in agreement with our results. DMI has also responded to RPL in some cases in mid-lactation (41), but not in others (42, 43). No such response was observed in lactating and dry cows (14, 44, 45). DMI has previously been reported to increase with supply RPM pre-calving (16, 46) and post-calving (33), similar to what was observed in our study. However, DMI responses to RPM were not detected pre-partum (47). In contrast to the present study, no response of DMI in transition cows continually consumed RPM and RPL before or after calving (15).

### Effects on Energy Balance and Body Condition Score

In our study, the response for feeding RPAA was matched with improved EB that is driven by the increase of DMI leading to 5, 5, and 13 kg/d greater ECM for cows supplemented with RPM or RPL or RPML, respectively, and this maintained BCS post-calving. Osorio et al. (33) showed that RPM cows need greater nutrients, particularly during post-calving, to compensate for high milk yield, agreeing with this work. However, pre- and post-calving EB was not affected by the provision of RPL to transition cows (19). The changes in BCS reflect the EB change observed in cows (48). In the current study, feeding dairy cows RPAA resulted in increased DMI and improved BCS, which minimized the changes in BCS at d 21 post-calving compared to d 21 pre-calving in this study. Dissimilarly, providing RPM pre-calving (14, 33, 49), or RPL did not affect BCS of transition cows (40, 50).

### Effect on β-Hydroxybutyrate Concentrations

In the current study, reduced BHBA concentrations with AAs supply are explained by the increased DMI, leading to improved EB, which could have reduced LCFA release from adipose tissue and hepatic conversation of FA to BHBA. During negative EB, there is a large uptake of adipose tissue–derived FA by the liver, which would have resulted in incomplete oxidation of NEFA and, consequently, elevated ketone bodies (10). Excessive adipose tissue mobilization results in incomplete hepatic oxidation and release of ketone bodies (10, 51), which was in the lower subclinical thresholds for cows fed RPML in the present study. Supply RPAA to cows during the transition period had varied effects on β-hydroxybutyrate. For instance, a previous study conducted by our laboratory (40), post-calving BHBA decreased when RPL was continually fed pre- and post-calving (40) or when RPM and RPL were supplemented in early lactation (15); however, post-calving BHBA was not affected by feeding RPL pre-calving (19). The decrease in pre-calving BHBA was only observed when RPL was provided pre-partum (19), in contrast to this experiment.

Another reason for the lower β-hydroxybutyrate with RPAA supply suggests that RPM and RPL might have improved hepatic lipid metabolism and increased carnitine bioavailability. Osorio et al. (33) found a tendency for decreasing the occurrence rate of clinical ketosis when RPM and HMBi were provided to transition cows suggesting that supplied Met might have improved hepatic lipid metabolism, similar to our findings. Lipotropic agents such as Met or choline assist in lipid export from the liver by stimulating VLDL formation (10). Consequently, that response may lead to a decrease in hepatic TAG accumulation and formation of ketone bodies (10, 52), which may assist in decreasing BHBA in the current study due to RPAA supply.

### Effects on Milk Production and Composition

In the present study, adequate dietary MP and AA balance have essential roles in improving milk protein content and yield. Similarly, milk protein outputs have been reported to increase by supply RPM to transition and dairy cows (33, 53) or a tendency for an increase in beef cows (20); however, this has not always been observed (9, 15). Authors noted that milk protein yield improves with increased MP (19, 54), which was revealed in the current study, and milk protein is influenced in direct proportion to the adequacy of Met in MP (3). Balancing the AA profile will probably alter milk protein in comparison with milk production (3, 15, 55). The supply of the IAA during the far-off dry period before the supplementation of RPAA during the close-up and its relative to cow's requirements may also affect the degree of response. The deficiency in IAA may also influence animals' response, animals likely should respond even if they were not deficient, but it also depends on how deficient the other IAAs are.

Protein supply is considered the limiting factor for the absorption of AAs in the mammary gland, which will be determined the yield of protein, lactose, and consequently milk production, as milk yield is a derivative of these components (56, 57). In the current study, increase in the supply of protein would increase protein yield and increase milk production and the required amount for lactose synthesis. The most limiting IAA for milk protein yield is Met, Lys, and, probably, His. Milk protein responses to Met are properly documented, and responses to Lys and His are somewhat more changeable but have often been observed (58, 59). Besides Met, Lys, and His, it shows that other two IAA of leucine (Leu) and isoleucine (Ile) are also contributed to driving milk protein production (60, 61). In the current study, milk fat percentage and yield are likely increased due to more available energy. In contrast, when energy is limiting, milk fat yield is possible to decrease. Notably, cows in the CON group are the most affected by this due to low DMI, resulting in less available energy, which was resulted in the least yield of milk fat.

By meeting or exceeding MP pre-calving, cows in the current study could easily adapt to post-partum more efficiently, as indicated by increased DMI, the main driver of milk yield. Supporting this, dairy cows fed MP-insufficient diets supplied with RPM, Lys, and His had milk yields that did not differ from cows provided MP-adequate diets (44), suggesting that providing RPAA- to MP-insufficient diets could be corrected for the deficiency. Several studies have demonstrated increased milk yield with RPM or RPL supply during the transition period (16, 19, 33, 62), in agreement with this study. Response to RPL has also been observed in later lactation cows (53, 63), but this response is not universal (15, 64–66).

The greater milk yield response to supplemental RPAA in this study may also be due to increased efficiency of absorbed AA use (in addition to providing adequate MP). Milk lactose and protein increased with AA supply, resulting in the use of a greater proportion of the unsupplemented IAA for milk protein. Junior et al. (53) reported that increased efficiency of absorbed AA increases milk yield in high-producing dairy cows (53). There are several reasons for the variations in milk production and constituents in response to RPAA supplementation; it is explained by the variability of protection methods for RPAA, which would have resulted in differing bioavailability, which may have accounted for differences in results between studies; the differences in the basal diets feedstuff, the milk production level, and the metabolizable amount of Met and Lys g/MP. In addition, the experimental length and animal type, lactation stage, farm management, and cows' comfort may also affect animal response to RPAA supply.

In the current study, RPAA supply to transition cows may positively affect udder health, as indicated by the lowered milk SCC. This improvement may be explained by that the RPAA plays an important role in improving immune status in transition cows through enhanced phagocytosis and oxidative functions, as reported when RPM was fed to dairy cows pre-calving and post-calving continuously (62). Many reasons can cause an increase in milk SCC; one of these, the inflammation in the mammary gland tissues (67), cow productivity, health status, lactation, lactation phase, and animal breed (68). The decrease of MUN observed in this study by providing RPAA to transition cows is indicative of greater efficiency of nitrogen use and an improvement in AA efficiency due to increase milk protein output. MUN decreased by feeding RPAA of either Met, Lys, Leu, and the combination to mid-to-late lactation dairy cows (69), or RP Met, THR, Ile, and Leu to mid-lactation (70), similar to what observed in the present study, but not in others (19, 65).

## Conclusions

In the current study, from the practical side, a continual supply of a combination of RPL and RPM throughout the transition period increased DMI and ECM production as well as improved EB. From the health side, the average β-hydroxybutyrate concentrations during the 3-wk post-calving period for cows that supplied either RPL or RPM were at the lower thresholds range of subclinical ketosis, whereas cows fed a combination was in the normal range of β-hydroxybutyrate concentrations. The results from the present study suggested that continual feeding RPL and RPM to transition cows is a practical strategy to improve milk production and performance efficiency and the health of perinatal dairy cows.

## Data Availability Statement

The raw data supporting the conclusions of this article will be made available by the authors, without undue reservation.

## Ethics Statement

The animal study was reviewed and approved by the Animal Care and Use Committee of the Institute of Animal Science, Chinese Academy of Agricultural Sciences, Beijing, China, approved the study protocol (No. IAS20180115).

## Author Contributions

SE: conceptualization, conducted the experiment, laboratory analysis, writing—original draft, and formal analysis. HW: conducted the experiment. ZW: investigation. MH: review and editing. DB: conceptualization, review and editing, investigation, supervision, project administration, and funding acquisition. All authors contributed to the article and approved the submitted version.

## Funding

This research was supported by the Key Research and Development Program of the Ningxia Hui Autonomous Region (2021BEF02018), the International Atomic Energy Agency Technical Co-operation and Assistance Programme (No. CPR5025), the Agriculture Science and Technology Innovation Program (ASTIP-IAS07-1), Chinese Academy of Agricultural Science and Technology Innovation Project (CAAS-XTCX2016011-01), and Beijing Dairy Industry Innovation Team (BAIC06-2021). SE was supported by a Ph.D. scholarship from the Graduate School of Chinese Academy of Agricultural Sciences (GSCAAS, Beijing, China).

## Conflict of Interest

The authors declare that the research was conducted in the absence of any commercial or financial relationships that could be construed as a potential conflict of interest.

## Publisher's Note

All claims expressed in this article are solely those of the authors and do not necessarily represent those of their affiliated organizations, or those of the publisher, the editors and the reviewers. Any product that may be evaluated in this article, or claim that may be made by its manufacturer, is not guaranteed or endorsed by the publisher.
